# Effect of Leisure Activity Participation on Leisure Attitude, Recreational Specialization, Leisure Satisfaction, and Intention to Re-Participate in South Korea

**DOI:** 10.3390/bs15030372

**Published:** 2025-03-16

**Authors:** Byoungwook Ahn

**Affiliations:** Department of Leisure Marine Sports, Seosan Campus, Hanseo University, Seosan-si 31692, Republic of Korea; bwahn75@hanseo.ac.kr

**Keywords:** leisure attitude, recreation specialization, leisure satisfaction, intention to re-participate, leisure activity

## Abstract

The importance of leisure in post-COVID-19 society has been underscored by the pandemic, illustrating the need to view leisure not merely as an option but as an essential component of a fulfilling life. This study investigates whether leisure attitudes, considered a novel perspective or belief regarding leisure, along with leisure activities, recreational specialization (the process of becoming proficient in particular leisure activities), and satisfaction derived from these experiences, prompt continued participation. The study surveyed 259 adults, both men and women, residing in Seoul, Gyeonggi, and Chungcheong Province who had engaged in leisure activities for over a year. Data were analyzed using the SPSS 21 program for frequency, reliability, and correlation analyses, while confirmatory factor analysis and structural equation modeling were performed with the AMOS 18.0 program. The research yielded the following findings: Firstly, the leisure attitudes of participants significantly influenced their leisure satisfaction. Secondly, their leisure attitudes did not influence recreational specialization. Thirdly, their attitudes did not affect their intention to re-participate. Fourthly, recreational specialization significantly affected both leisure satisfaction and intention to re-participate. Lastly, leisure satisfaction significantly influenced the intention to re-participate. With the evolving perceptions of leisure post-pandemic, there is a need for policies and infrastructure that support the sustainable engagement of leisure activity participants.

## 1. Introduction

We live in a society rich in leisure culture. Since the early 2000s, the expansion in leisure time has integrated into our lives. The productive utilization of leisure not only shapes the quality of an individual’s life but also acts as a criterion for cultural norms (Ha, 2006). Particularly during the COVID-19 pandemic, the significance of utilizing leisure time has been accentuated. As the virus proliferated, social distancing curtailed face-to-face interactions, while contactless and individual activities driven by digital and data interactions have thrived. This trend is likely to establish a new standard rather than a temporary shift, featuring developments such as online education, remote work, virtual conferences, online banking, telehealth, e-commerce, automated sales, autonomous taxis, digital voting, and virtual reality experiences (K. Y. Kim, 2022). Given that leisure has become a pivotal element of human life both before and after COVID-19, leisure researchers have scientifically explored the dynamics of leisure involvement and human behavior (M. J. Lee & Lee, 2010). Leisure has evolved from a “choice” to a critical factor that influences “how it is utilized to enhance life’s enjoyment and significance”. Han and Sa (2022) investigated the relationship between leisure attitudes, stress-related growth, and quality of life in a social distancing environment due to COVID-19 and found that leisure attitudes promote stress-related growth, and stress-related growth plays a role in improving quality of life. According to Yang et al. (2023), it is important to develop various programs to promote positive attitudes toward leisure and to enable us to participate more actively in leisure activities. Tian et al. (2022) also said that research is needed to reexamine the role of leisure activities in the changed environment after the COVID-19 pandemic.

This study also examines shifts in perceptions of leisure post-COVID-19 among leisure activity participants, the association between leisure engagement and recreational specialization, and the link between satisfaction derived from leisure experiences and the intention to re-participate.

### 1.1. Leisure Attitude

Leisure attitude can be described as a mental orientation toward leisure, incorporating personal attributes like motivation and beliefs regarding leisure engagement (Iso-Ahola, 1980). Leisure attitude exerts a favorable influence on the relationship with leisure behaviors; the more positive a participant’s attitude, the greater their propensity to continue engaging in leisure activities. Thus, it is acknowledged as a critical factor in leisure involvement (Godin & Shephard, 1986; Manfredo et al., 1992). Leisure attitude comprises cognitive, emotional, and behavioral components, and it is evident that the formation of leisure attitudes fosters recreational specialization, thereby enhancing leisure satisfaction levels (Y. J. Park et al., 2015). According to a study by Y. S. Choi and Cho (2012), leisure attitude impacts various facets of life, and positive attitudes and interest in leisure improve satisfaction with leisure activities (Kang, 2011). Moreover, a positive leisure attitude enhances the continuity of physical activities (Shin & Choi, 2020), and it directly shapes the intention to sustain leisure sports participation (D. H. Kim et al., 2009). A study by Yoo (2022) suggests that attitudes toward leisure and satisfaction with leisure policies can promote the happiness of caregivers of young children. And, as interest in happiness and quality of life has increased in Korea recently, the importance of leisure is increasingly being emphasized. Han and Sa (2022) investigated the relationship between leisure attitudes, stress-related growth, and quality of life in a social distancing environment due to COVID-19 and found that leisure attitudes promote stress-related growth, and stress-related growth plays a role in improving quality of life. According to Yang et al. (2023), leisure attitude and perceived value were found to mediate the relationship between leisure motivation and happiness. In addition, there is a need to understand in depth the relationship between leisure activities and happiness, and more extensive analysis is needed in future sports and leisure-related research. Given these insights, this study hypothesizes that leisure attitude significantly affects recreational specialization, leisure satisfaction, and the intent to re-participate.

### 1.2. Recreational Specialization

Recreational specialization is defined as a continuous process whereby participants in recreation evolve from novices to experts over time, illustrating the “process” of leisure engagement (Bryan, 1977). Even within the same leisure activity, individual differences manifest at various specialization levels. This specialization shapes group dynamics, varying leisure experiences, and levels of satisfaction (Seo & Lee, 2008). Understanding these disparities enables the explanation and prediction of behaviors and patterns among leisure activity participants. Recreational specialization encompasses the development of specific behaviors, skills, and commitments (Scott & Shafer, 2001), with time invested in acquiring skills or knowledge in particular leisure activities frequently utilized as a metric (Scott & Godbey, 1994). According to Randler (2023), participants in leisure activities differ in their level of participation, specialization, and immersion, and recreation specialization can be understood as a continuous spectrum from beginners to highly skilled experts. Tian et al. (2022) found that recreational specialization and self-efficacy positively influenced participants’ immersive experiences in long-distance running. Additionally, recreational specialization, self-efficacy, and flow experience were found to have significant positive correlations with life satisfaction. Previous studies have documented that recreational specialization has a positive effect on leisure satisfaction and the intention to re-participate.

### 1.3. Leisure Satisfaction

Leisure satisfaction pertains to the degree of satisfaction participants derive from their leisure experiences (Newman et al., 2014). It represents subjective satisfaction derived from either passive or active engagement in leisure activities during leisure time (Sirgy et al., 2017). Research indicates that social, emotional, and environmental dimensions of leisure satisfaction positively influence the intention to re-participate (S. Cho & Kim, 2014). It has also been observed that diversified programs focusing on educational content, physical strength enhancement, and infrastructure development boost leisure satisfaction and re-participation intentions. Additional studies suggest that frequent engagement in leisure activities provides increased opportunities for social interactions and skill development, thereby enhancing well-being (Kuykendall et al., 2015).

Therefore, this study aims to explore the leisure attitudes, recreational specialization, leisure satisfaction, and re-participation intentions of participants in leisure activities in a post-COVID-19 society. Acknowledging the enhanced significance of leisure following COVID-19, there has been a heightened interest in “how enjoyable life can be”. According to Osoch et al. (2025), people generally travel to increase their life satisfaction. In the Netherlands, a longitudinal analysis was conducted over an eight-year period to study the relationship between leisure travel and life satisfaction. According to Zhao et al. (2025), research results have been published recently worldwide that show that active leisure activities improve the mental health of participants, and research is being conducted on the relationship between leisure participation and mental health. Tian et al. (2020) empirically demonstrated that serious leisure can improve subjective happiness through leisure satisfaction. And, to design leisure activities more effectively and increase participant satisfaction, it was said that factors that enhance leisure satisfaction should be considered. The objective of this study is to offer foundational data to support the expansion of leisure infrastructure and the formulation of leisure policies.

### 1.4. The Hypothesis of the Study

To analyze the purpose of this study, the following research hypothesis was established. First, leisure activity participants’ leisure attitudes will affect recreation specialization, leisure satisfaction, and intention to re-participate. Second, leisure activity participants’ recreation will affect leisure satisfaction and intention to re-participate. Third, the leisure satisfaction of leisure activity participants will affect their intention to re-participate.

## 2. Materials and Methods

### 2.1. Subject of This Study

The subjects of this study were selected as adult men and women engaged in various leisure activities. The sampling method employed was purposive sampling, specifically targeting adults residing in Seoul, Gyeonggi-do, and Chungcheong-do. Purposive sampling is a method in which researchers recruit samples by selecting a specific group to analyze the purpose of the study. The researcher selected adult men and women participating in leisure activities as the target population and conducted an online survey. This study was conducted through an online survey. Participants were selected based on their participation in leisure activities (hobbies, entertainment, sports, socializing, travel) for a period exceeding one year. The researcher searched for Internet clubs on portal sites, and the purpose of the study was to explain to club leaders to secure their consent. After obtaining consent from the club management, we received the email addresses of the club members and sent them a research description and questionnaire via email. Research participants participated voluntarily of their own free will.

### 2.2. Measurement

A questionnaire served as the research instrument, covering socio-demographic characteristics of the participants and various research variables, including leisure attitude, recreation specialization, leisure satisfaction, and intention to re-participate. Survey items were derived from domestic and international dissertations and scholarly journals to better align with the research objectives. The measurement tools are detailed as follows:

#### 2.2.1. Socio-Demographic Characteristics

Socio-demographic variables that could influence participation in leisure activities were considered, encompassing four categories: gender, age, residence, and the type of leisure activity.

#### 2.2.2. Leisure Attitude

Leisure attitude factors were measured using items developed by M. J. Lee (2006), comprising cognitive (6 items), emotional (7 items), volition (3 items), and behavioral (3 items) dimensions, culminating in a total of 19 items. Assessment was conducted using a 5-point Likert scale ranging from “Strongly Disagree (1)” to “Strongly Agree (5)”. Representative items include “Leisure activities enhance happiness (cognitive)”, “I believe leisure activities are beneficial (emotional)”, “I invest significant time and effort in leisure activities (volitional)”, and “I participate in seminars related to leisure activities (behavioral)”.

#### 2.2.3. Recreation Specialization

The recreation specialization scale from M. J. Lee et al. (2011) was utilized to ascertain recreation specialization levels. This scale includes four dimensions: information (3 items), skill (3 items), experience (3 items), and investment (3 items), totaling 12 items. Evaluation was accomplished using a 5-point Likert scale from “Strongly Disagree (1)” to “Strongly Agree (5)”. Sample items consist of “I possess comprehensive knowledge (information)”, “I am capable of elucidating technical principles (skill)”, “I regularly engage in leisure activities (experience)”, and “I invest substantially in gear for leisure activities (investment)”.

#### 2.2.4. Leisure Satisfaction

Leisure satisfaction was measured with indicators from the Korean adult leisure satisfaction scale, developed by Ahn (2009), featuring sub-factors such as self-development (3 items), stress relief (4 items), skill enhancement (4 items), health enhancement (3 items), and interpersonal relationship improvement (3 items), accumulating to 17 items. The scale employed was a 5-point Likert scale extending from “Strongly Disagree (1)” to “Strongly Agree (5)”. Exemplary items include “Leisure assists in personal development (self-development)”, “Leisure activities are conducive to stress alleviation (stress relief)”, “Leisure activities contribute to physical well-being (health promotion)”, “Leisure requires relevant skills (skill improvement)”, and “Leisure enables new social connections (interpersonal relationship enhancement)”.

#### 2.2.5. Intention to Re-Participate

The scale for measuring the intention to re-participate incorporated items from S. Park (2008) and consisted of 5 items as a single factor. The intention to re-participate was assessed using a 5-point Likert scale ranging from “Strongly Disagree (1)” to “Strongly Agree (5)”.

### 2.3. Validity and Reliability

#### 2.3.1. Validity

To ascertain the validity of the research instruments, confirmatory factor analysis (CFA) was employed. CFA, a method for validating validity, tests whether measurement variables accurately depict the intended concept of latent variables (Hong, 2007). This study utilized the standard Chi-square (*x*^2^*/df*) suggested by Carmines and McIver (1983), the Comparative Fit Index (CFI) by Bentler (1990), and the Non-Normed Fit Index (NNFI), known as the Tucker–Lewis Index (TLI) in AMOS 18.0, by Bentler and Bonett (1980). Additionally, the Root Mean Square Error of Approximation (RMSEA) by Steiger and Lind (1980) indicated that *x*^2^ exceeds 0.05 (G. Kim, 2011).

The results of the CFA in this study indicated that the fit indices *x*^2^*/df*, CFI, TLI, and RMSEA for each factor structure were all appropriate, as illustrated in (Table 1). Analysis of the fit indices by research variable revealed appropriate values for the leisure attitude measurement model, with *x*^2^*/df* = 2.658, CFI = 0.954, TLI = 0.935, and RMSEA = 0.067. For the recreation specialization measurement model, the fit indices reported were *x*^2^*/df* = 2.601, CFI = 0.958, TLI = 0.937, and RMSEA = 0.066. In the leisure satisfaction measurement model, the fit indices registered were *x*^2^*/df* = 2.848, CFI = 0.969, TLI = 0.950, and RMSEA = 0.071. Given that all indices for leisure attitude, recreation specialization, and leisure satisfaction met the model fit criteria, the models were considered valid for further analysis.

#### 2.3.2. Reliability

To evaluate the reliability of the study variables, we calculated Cronbach’s alpha between the overall and sub-factor-related verification levels. Reliability was confirmed with the overall reliability of questions exceeding 0.5 and sub-factor reliability exceeding 0.6. Specifically, the reliability of leisure attitude’s sub-factors was analyzed as 0.882, 0.836, 0.767, and 0.751. The reliability of recreation specialization was 0.884, 0.850, 0.656, and 0.595, whereas leisure satisfaction was 0.837, 0.852, 0.828, 0.906, and 0.876. The reliability of the intention to re-participate was analyzed at 0.894 (Table 2).

### 2.4. Data Analysis Process

The data analysis for this study was conducted using SPSS 21.0 and AMOS 18.0. Frequency analysis was carried out on the socio-demographic variables of the study participants. Confirmatory factor analysis was employed to verify the validity of the research variables, while reliability was assessed through Cronbach’s α to determine internal consistency. The correlation among research variables was verified using a bivariate correlation technique. A structural equation model (SEM) was utilized to examine structural relationships among the research variables.

### 2.5. Study Ethics

All research procedures were reviewed and approved by the Institutional Review Board of Hanseo University (HUIRB-2024-0319-01). The research comprised the guidelines outlined in the Declaration of Helsinki. Participants gave their consent to join the study, and the purposes and duration of the research were explained to them. All participants authorized the researchers to use their personal information obtained from questionnaires for research purposes and were informed that they could withdraw from the study at any time.

## 3. Results

### 3.1. Participants

The demographic analysis of the research participants revealed the following results: there were 143 male and 116 female participants. The age distribution was as follows: 57 participants were in their 20s, 71 in their 30s, 69 in their 40s, and 62 were aged 50 and above. Regarding the region of residence, 89 participants came from Seoul, 92 from Incheon, and 78 from Chungcheong-do. Participants were involved in various types of leisure activities: 74 engaged in hobbies, 43 in entertainment, 62 in sports, 41 in cultural activities, and 39 in travel (Table 3).

### 3.2. Correlation

Prior to verifying the structural equation model, confirmatory factor analysis was performed to confirm the homogeneity of each research variable’s scale. Subsequently, a correlation analysis was conducted to examine the relationships between the variables (Table 4). The analysis showed that leisure attitude had no significant correlation with recreational specialization (*r* = 0.053) and re-participate intention (*r* = 0.046), yet it was significantly correlated with leisure satisfaction (*r* = 0.317). Recreational specialization correlated significantly with both leisure satisfaction (*r* = 0.532) and intention to re-participate (*r* = 0.461). Leisure satisfaction was significantly correlated with the intention to re-participate (*r* = 0.384). Since all correlation coefficients between variables were below the multicollinearity threshold of 0.80, multicollinearity was considered not to be an issue.

### 3.3. Model Fit

The results of the model fit indices were as follows: *χ*^2^ = 267.378, CFI = 0.945, TLI = 0.924, RMSEA = 0.061. These values indicate that all fit indices met the necessary thresholds, confirming that there were no issues in hypothesis validation (Table 5).

### 3.4. Hypothesis Verification

The structural equation model was utilized to explore the relationships between leisure attitude, leisure satisfaction, recreational specialization, and intention to re-participate among participants of leisure activities. The findings are as follows (Table 6). Hypothesis 1-1: Leisure attitude did not significantly influence recreational specialization (*β* = 0.025, *t* = 0.707, *p* > 0.05). Hypothesis 1-2: Leisure attitude positively affected leisure satisfaction (*β* = 0.161, *t* = 1.855, *p* < 0.01). Hypothesis 1-3: Leisure attitude did not significantly impact the intention to re-participate (*β* = 0.035, *t* = 0.463, *p* > 0.05). Hypothesis 2-1: Recreational specialization had a significant positive effect on leisure satisfaction (*β* = 0.399, *t* = 4.011, *p* < 0.001). Hypothesis 2-2: Recreational specialization significantly influenced the intention to re-participate (*β* = 0.254, *t* = 3.043, *p* < 0.001). Hypothesis 3: Leisure satisfaction significantly influenced the intention to re-participate (*β* = 0.046, *t* = 1.911, *p* < 0.01).

## 4. Discussion

### 4.1. The Relationships Between Leisure Attitude, Recreational Specialization, Leisure Satisfaction, and Intention to Re-Participate

The leisure attitudes of participants in leisure activities positively influenced their leisure satisfaction; however, these attitudes did not impact on recreational specialization or the intention to re-participate. This indicates a potential gap in understanding the importance of leisure attitude for sustained leisure activity participation. The COVID-19 pandemic might have intensified this issue as individuals prioritized stability in daily life over leisure pursuits. Leisure activities are individually chosen and engaged in during free time, with recreational specialization involving continuous engagement and encompassing knowledge, skills, investments, and experiences pertinent to leisure pursuits. Meanwhile, the intention to re-participate reflects the decision to continually engage in a particular leisure activity.

Although leisure is considered important, personal stability takes precedence. This study finds that while leisure attitudes do not significantly affect either recreational specialization or the intention to re-participate, they do positively impact leisure satisfaction. Despite prioritizing life stability, participants tend to prefer small-scale and accessible leisure activities such as hobbies, entertainment, and travel, which require neither specialized skills nor substantial expenses. This likely explains why leisure attitudes positively affect leisure satisfaction.

According to the study by Y. Lee and Kim (2020), the finding that cognitive immersion in rhythmic exercise had no effect on recreation specialization partially supports the results of this study. S. Choi (2021) identified a moderating effect of income on the relationship between leisure consumption and satisfaction, offering deeper insights into leisure capital, time, and the utility of leisure activities, which corroborates the findings of this study. K. Kim (2023) observed that the emotional and behavioral attitudes of the MZ generation positively affect psychological, social, and environmental leisure satisfaction, while cognitive attitudes were found to influence social and environmental leisure satisfaction. According to Osoch et al. (2025), people generally travel to increase their life satisfaction. In the Netherlands, a longitudinal analysis was conducted over an eight-year period to study the relationship between leisure travel and life satisfaction. Yoo (2022) suggests that a positive attitude toward leisure and satisfaction with leisure policies can increase satisfaction with leisure activities, thereby enhancing happiness, and may provide useful implications for establishing future leisure policies and improving the family care environment. According to Wen (2025), it is necessary to study the weight of the four criteria of recreational resource diversity, service quality, price strategy, and environmental quality and the tourists’ intention to revisit.

### 4.2. The Relation Between Recreation Specialization, Leisure Satisfaction, and Intention to Re-Participate

The recreational specialization of leisure activity participants significantly influenced both leisure satisfaction and the intent to re-participate. Recreational specialization entails continued involvement in specific leisure activities, advancing from beginner to enthusiast levels. The study suggests that the recreational specialization of participants in sports-related leisure activities enhances leisure satisfaction and the intent to re-participate. Sports-related leisure activities encourage sustained involvement from beginners to enthusiasts, positively impacting leisure satisfaction in areas such as personal growth, health, and interpersonal relationships, thereby promoting a positive influence on the intention to re-participate.

According to H. Cho et al. (2023), recreation specialization involves a persistent effort to enhance skills and knowledge pertaining to one’s leisure activities, exhibiting a spectrum of behavioral patterns from basic participation to specialized engagement. H. Kim et al. (2013) asserted that leisure satisfaction is not just determined by participation in leisure activities but also profoundly influenced by the degree of specialization. According to the study by M. Lee and Hwang (2014), recreation specialization enhances leisure satisfaction through elevated levels of skill, proficiency, recognition of the value of leisure, confidence, and a sense of accomplishment. The study by M. Kim et al. (2023) discovered that recreation specialization not only directly and positively impacts leisure satisfaction but also shows a distinct moderating effect on leisure satisfaction through its interaction with conspicuous leisure consumption tendencies. According to Zhao et al. (2025), the mental health of hikers was positively affected by attractiveness, centrality, and self-expression, and self-efficacy and social support played a moderating role in the relationship between self-expression, attractiveness, and mental health. According to Randler (2023), participants in leisure activities differ in their level of participation, specialization, and immersion, and recreation specialization can be understood as a continuous spectrum from beginners to highly skilled experts. According to Wu et al. (2021), it is important to develop various programs to promote positive attitudes toward leisure and to enable us to participate more actively in leisure activities.

### 4.3. The Relation Between Leisure Satisfaction and Intention to Participate

Participant satisfaction in leisure activities positively influences their intention to re-participate. Leisure satisfaction pertains to the emotional experiences of participants during such activities. Participation in hobbies, entertainment, travel, and sports generates enjoyment and interest, contributing to greater satisfaction and thereby enhancing the intention to continue these activities. Notably, after approximately two years of constrained leisure opportunities due to COVID-19, participants reported increased leisure satisfaction upon resuming these activities, which positively affected their re-participation intentions.

According to the study by Song and Ahn (2023), individuals involved in sports activities like soccer, badminton, and swimming demonstrate elevated leisure satisfaction, which includes benefits such as stress relief, health improvement, and skill development, fostering their continued re-engagement. Research by Kuykendall et al. (2015) and Newman et al. (2014) revealed that regular participation in leisure activities offers enhanced opportunities for skill and knowledge acquisition pertinent to these activities and promotes positive social interactions, thereby making frequent engagement in leisure activities an effective strategy for boosting well-being. Moreover, S. Cho and Kim (2014) identified that the sub-factors of leisure satisfaction—social, emotional, and environmental—positively impact re-participation intentions. They recommended the implementation of diversified program levels, the development of educational and physical fitness opportunities, facility expansion, the creation of better infrastructure for easier access to snowboarding, and the fostering of environments that support interpersonal relationships, all aimed at augmenting leisure satisfaction among snowboarding enthusiasts and increasing their likelihood to re-engage.

According to Han and Sa (2022), the relationship between leisure attitudes, stress-related growth, and quality of life in a social distancing environment due to COVID-19 was identified, and leisure attitudes promoted stress-related growth, and stress-related growth played a role in improving quality of life. According to Tian et al. (2020), it was empirically proven that serious leisure can improve subjective happiness through leisure satisfaction. And, to design leisure activities more effectively and increase participant satisfaction, it was said that factors that enhance leisure satisfaction should be considered.

## 5. Conclusions

The significance of leisure in post-COVID-19 society has been underscored by the pandemic, redefining leisure not merely as a “choice” but as an essential component of a fulfilling life. This study seeks to determine whether attitudes toward leisure, viewed as a novel perspective or belief about leisure, together with leisure activities, recreational specialization (the process of becoming an expert in particular leisure pursuits), and the satisfaction derived from leisure experiences contribute to sustained participation.

This study was able to reaffirm the importance of leisure activities while experiencing extreme environments such as COVID-19. It also contributed to a deeper understanding of the relationships among leisure attitudes toward leisure activities, recreation specialization, leisure satisfaction, and intention to re-participate. However, the fact that leisure attitudes do not directly affect re-participation intentions suggests that to continuously induce individuals’ leisure participation, practical support policies and programs are needed in addition to attitude improvement. Additionally, for dynamic leisure activities such as sports, participation persistence is high, so there is a need to provide specialized training programs and low-cost participation opportunities within the community. To increase accessibility to leisure activities, expansion of public leisure facilities and development of programs that can accommodate people of different age groups are required.

### 5.1. Limitations of the Study

This study presents several limitations and suggestions for future research. Firstly, the study was restricted to adult males and females engaged in leisure activities in Seoul, Gyeonggi, and Chungcheong provinces, which may not adequately represent the entire adult population of South Korea. Secondly, although there are about 108 types of leisure activities in Korea, this study was limited to specific categories such as hobbies, entertainment, sports, travel, and socializing, thus not fully encapsulating all leisure activity types. And the longitudinal design would be more appropriate for the research questions rather than the cross-sectional design in the study; however, the reviewer recognizes the challenges associated with performing longitudinal studies on leisure-based populations. Another limitation is the broad variables “hobbies” and “sports”. These encompass many leisure and recreation options, and the high response number is of note here. Division of these into more specific leisure options (e.g., creative arts or nature-based recreation) could affect the results of this study. Lastly, some of the sub-scale reliability is marginal (e.g., experience and investment). The use of these scales on a specific population for which the original scale may not have been developed.

### 5.2. Directions for Future Research

Building on the research methodology, the following suggestions for future research are proposed: Initially, the study was conducted using a quantitative approach. Future research employing qualitative methods could uncover insights that are not accessible through quantitative techniques. Secondly, among the measurement tools used in this study, the leisure satisfaction scale was developed internationally. Developing culturally and emotionally relevant measurement tools specific to Korea may yield novel and more precise research outcomes.

## Figures and Tables

**Table 1:** The results of validity.

	*x* ^2^	*x* ^2^ */df*	CFI	TLI	RMSEA
Leisure attitude	196.698	2.658	0.954	0.935	0.067
Recreation specialization	114.428	2.601	0.958	0.937	0.066
Leisure satisfaction	239.257	2.848	0.969	0.950	0.071

**Table 2:** The result of reliability.

Variable		Number of Questions	Cronbach’ α
Leisure attitude	Emotional	6	0.882
	Cognitive	7	0.836
	Behavioral	3	0.767
	Volitional	3	0.751
Recreation specialization	Information	3	0.884
	Skill	3	0.850
	Investment	3	0.656
	Experience	3	0.595
Leisure satisfaction	Self-development	3	0.837
	Stress relief	4	0.852
	Health	3	0.828
	Skill improvement	4	0.906
	Interpersonal relationship enhancement	3	0.876
Intention to re-participate		5	0.849

**Table 3:** The result of the socio-demographic analysis of the study subjects.

Items		N	Items		N
Gender	Male	146	Age	20s	57
	Female	116		30s	71
Leisure activity type	Hobbies	74		40s	69
	Entertainment	43		50s over	62
	Sports	62	Region	Seoul	89
	Cultural	41		Incheon	92
	Travel	39		Chungcheong	78

**Table 4:** The result of the correlation analysis.

	1	2	3	4
Leisure attitude	1			
Recreation specialization	0.053	1		
Leisure satisfaction	0.317 **	0.532 ***	1	
Intention to re-participate	0.046	0.461 ***	0.384 **	1

** *p* < 0.01, *** *p* < 0.001.

**Table 5:** The results of model fit verification.

	*x* ^2^	*x* ^2^ */df*	CFI	TLI	RMSEA
Model fit	267.378	2.387	0.945	0.924	0.061

**Table 6:** The results of the hypothesis verification.

Hypothesis		Estimate	S.E.	C.P	
H 1-1	Leisure attitude → Recreation specialization	0.025	0.036	0.707	Unaccept
H 1-2	Leisure attitude → Leisure satisfaction	0.161	0.087	1.855 **	Accept
H 1-3	Leisure attitude → Intention to re-participate	0.035	0.075	0.463	Unaccept
H 2-1	Recreation specialization → Leisure satisfaction	0.399	0.100	4.011 ***	Accept
H 2-2	Recreation specialization → Intention to re-participate	0.254	0.084	3.043 ***	Accept
H 3	Leisure satisfaction → Intention to re-participate	0.046	0.050	1.911 **	Accept

** *p* < 0.01, *** *p* < 0.001.

## Data Availability

The anonymized data that support the findings of this study are available on request from the corresponding author. The data are not publicly available due to information that may compromise the participants’ privacy.
